# Empidinae (Diptera, Empididae) from Bulgaria with new records and descriptions of three new species

**DOI:** 10.3897/zookeys.785.26236

**Published:** 2018-09-19

**Authors:** Liliana Kanavalová, Štěpán Kubík, Miroslav Barták

**Affiliations:** 1 Department of Zoology and Fisheries, Faculty of Agrobiology, Food and Natural Resources, Czech University of Life Sciences Prague, Kamýcká 129, 165 00 Praha-Suchdol, Czech Republic Czech University of Life Sciences Prague Prague Czech Republic

**Keywords:** *
Empis
*, Europe, *
Hilara
*, new species, Palaearctic region, *
Rhamphomyia
*

## Abstract

Empis (Leptempis) rhodopensis Barták** sp. n.**, *Hilarabulgarica* Barták** sp. n.**, and Rhamphomyia (Amydroneura) stojanovae Barták** sp. n.** are described from Bulgaria. Altogether 32 species of Empididae are first reported from Bulgaria.

## Introduction

The Empididae is a large family of DipteraBrachycera with approximately 1300 species known from the Palaearctic region and almost 1000 of them belonging to three genera: *Empis* Linnaeus, *Hilara* Meigen, and *Rhamphomyia* Meigen.

The Empididae fauna of Bulgaria has never been systematically studied and the results of chance findings are scattered among several papers (e.g., Loew 1862; Beschovski 1973; Straka 1976; Chvála 1977, 2002, 2005a, b, 2008; Chvála and Wagner 1989; Ceianu 1992; Beschovski and Dzhambazov 1998; Dzhambazov 1998; Dzhambazov and Beschovski 2000; Çiftçi and Hasbenli 2008; Chvála and Merz 2009; Kustov and Shamshev 2011; Özgül and Civelek 2013; Hubenov 2015), but older records are generally not reliable. In our paper we provide numerous new records of the Empididae subfamily Empidinae from Bulgaria, including descriptions and illustrations of three new species.

General distributions of species are taken from Wyatt (2014) supplemented by recent checklist of Russian Empidoid flies (Shamshev 2016) and several revisions of *Hilara* (Chvála 2002, 2005a, b, 2008; Chvála and Merz 2009) and *Rhamphomyia* (Barták 2006a, b, 2007a, b).

## Materials and methods

The material studied originated from recent collections of authors (MB and ŠK) in Bulgaria (Rhodopes Mountains, Pirin Mountains, Central Balkan) and it is deposited in the collection of the Czech University of Life Sciences, Prague (**CULSP**). The material was collected by means of sweeping vegetation and yellow and white water pan traps, and stored in ethyl alcohol. Voucher specimens were selected and dried using methods described by Barták (1997).

Genitalia preparations and drawings: genitalia, together with the preceding 2–3 abdominal segments were removed from the rest of the body using small scissors and macerated in potassium hydroxide solution (approx. 10%) in small vials submerged in hot water for 1–2 hours. After neutralizing with 8% acetic acid (5 minutes), the genitalia were dissected in glycerine and photographed using an Olympus E-410 digital camera mounted on an Olympus BX51 compound microscope. Resulting images were edited with the computer software Quick Foto micro 2.3 provided with deep focus 3.1. Final images were a montage composed usually of 7–15 layers and were further edited with ®Adobe Photoshop. Images served as model for hand drawings, details were added directly observing objects.

The morphological terms used here follow Merz and Haenni (2000), Sinclair (2000), and Sinclair and Cumming (2006). All body measurements (including body and setae length) were taken from dry specimens (therefore the actual length may differ from that of fresh or wet-preserved material) by means of an ocular micrometre mounted on Nikon SMZ 1500 binocular microscope. Abbreviations: M2/d ratio = length of vein M_2_: greatest length of discal medial cell (discal cell); CuA1 ratio = length of apical: preapical sections of vein CuA_1_; lw: ww ratio = greatest length of wing (from basicosta to apex): greatest width of wing. Antennal segments were measured in 0.01 mm scale. Male body length was measured from antennal base to the tip of genitalia and female body length from base of antennae to the tip of cerci. Thoracic setae are counted on one side of body except scutellars.

Geographical coordinates were either found with GPS using map datum WGS-84 or were obtained from Google Earth.

## Descriptions of new species

### Empis (Leptempis) rhodopensis

Taxon classificationAnimaliaDipteraEmpididae

Barták sp. n.

http://zoobank.org/CB20F48C-4546-47A9-A714-624D63BA9230

Figs 1 , 2

#### Type material.

**HOLOTYPE** ♂, Bulgaria, Rhodopes Mountains, Pamporovo env., meadow, 41.65N, 24.73E, 1600 m, Barták, Kubík, 22.–24.vi.2016 (CULSP). **PARATYPES**: 4♂, 2♀, same data as holotype; 1♂ Bulgaria, Rhodopes Mountains, Sniezhanka peak, 1900m, hilltop, 41.637N, 24.680E, Barták, Kubík, 24.–25.vi.2016 (CULSP).

#### Diagnosis.

Large species of the E. (L.) rustica group with yellow coxae, very long labrum (2.5–2.6× head height), grey abdomen incl. venter, brownish yellow epandrial lamella, and spinose ventral part of hind femur.

#### Etymology.

The species epithet, *rhodopensis*, is derived from mountain range (Rhodopes Mountains) where the type material was collected.

#### Description.

**Figure 1.**
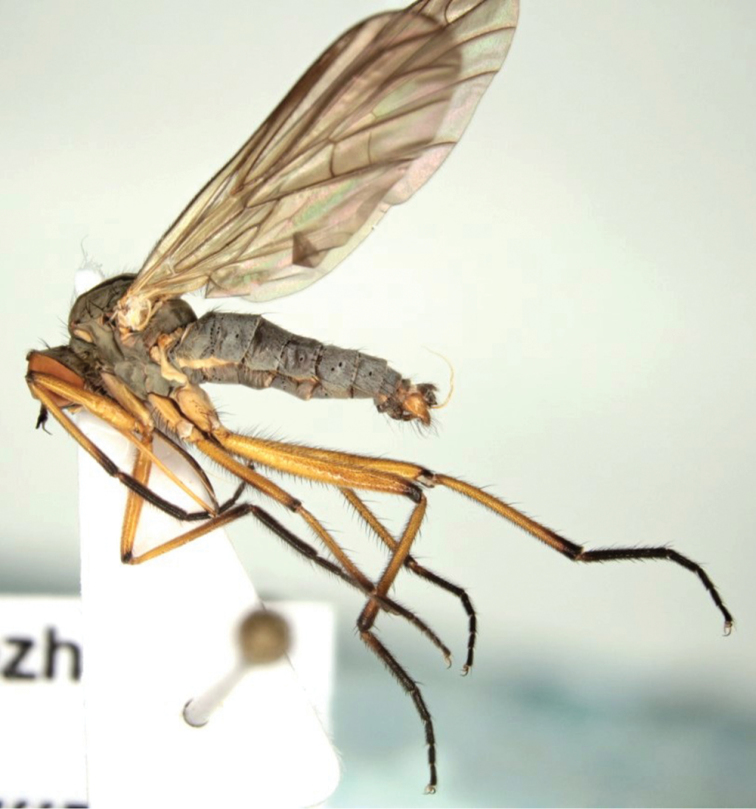
*Empisrhodopensis* sp. n. Habitus, male, lateral view.

**Male (Figure 1). Head** black, rather light grey microtrichose, holoptic, eyes meeting over long distance. Frons withsmall triangles just above antennae and below front ocellus without setae. Dorsal half of eye with distinctly larger facets than ventral half. Ocellar setae black, broken in all male specimens. Occiput with two rows of long setae dorsally, ventral part with irregularly arranged, long and fine yellow setae. Face approx. 0.35 mm broad ventrally, microtrichose with shiny narrow ventral margin, without setae. Clypeus shiny, gena very narrow, microtrichose. Palpus yellow, with several short black setae, subapical seta longest. Labrum yellow with brown base and apex, 2.5–2.6× longer than head height. Antenna black, both basal segments short setose; length of antennal segments (scape: pedicel: postpedicel: basal joint of stylus: last joint of stylus) = 0.18–0.21 mm: 0.1–0.11 mm: 0.39–0.43 mm: 0.04–0.05 mm: 0.21–0.3 mm.

**Thorax** black, light grey microtrichose; mesoscutum with three stripes down rows of acrostichal and dorsocentral setae brown in anterior view and almost velvety blackish-brown in posterior view, postalar callus yellowish. Chaetotaxy: antepronotum with row of 4–6 black setae and several pale setulae laterally, proepisternum with many fine pale setae, prosternum bare; acrostichals biserial and approx. 0.20 mm long (subequally long as distance between rows of acrostichals and dorsocentrals); dorsocentrals longer and stronger, irregularly 2–3-serial (in some specimens almost uniserial anteriorly) ending in 2–4 longer prescutellar pairs; 1 long postpronotal and several smaller setulae; one long posthumeral (presutural supraalar), 1–2 long intrahumeral(s) (presutural intraalars); notopleuron with 2–3 long and strong setae and with several additional setae anteriorly (sometimes one of them equally strong as posterior ones); two postsutural supraalars (and 0–2 finer setae in prealar position); one long postalar (and several very small black setulae); two pairs of long scutellars (outer pair smaller); laterotergite in some specimens with yellow setae, in others mixed with black setae (on anterior part of laterotergite). **Legs** including coxae yellow, tips of all femora and tibiae, and whole tarsi (except sometimes basal part of hind basitarsus) black, all setae black except some pale setulae anteriorly on fore coxa. Fore femur very short setose, with rows of antero- and posteroventral very fine setulae shorter than half of femur depth. Fore tibia short setose, in some specimens with one to several setulae dorsally shorter than tibia depth, posterior setosity rather dense. Mid femur with fine and medium dense anterodorsal setosity shorter than femur depth, posterodorsal setulae much shorter, anteroventral row of setae slightly shorter than femur depth, posteroventral setae slightly longer. Mid tibia with two ventral rows of setae slightly longer than tibia depth, dorsally with two rows of 4–5 setae up to twice longer than tibia depth. Hind femur short setose dorsally except several somewhat longer fine anterodorsal setae on apical part, posteriorly and posteroventrally rather densely covered with fine setae slightly longer than femur depth, anteroventrally and ventrally with irregularly arranged short spine-like setae. Hind tibia with short ventral ciliation, two rows of 4–5 setae dorsally slightly longer than tibia depth. Basitarsi of all legs slender and short setose, hind one with short spine-like setae ventrally. Comb at tip of hind tibia without longer seta. **Wing** membrane distinctly brown clouded, veins yellowish to blackish brown, axillary angle slightly acute; costal seta present, fine and rather short. Measurements: M2/d ratio = 1.0–1.2, CuA1 ratio = 1.4–1.6, lw:ww ratio = 3.2–3.5. Halter yellow, calypter light brownish with yellow margin and yellow fringes.

**Figure 2.**
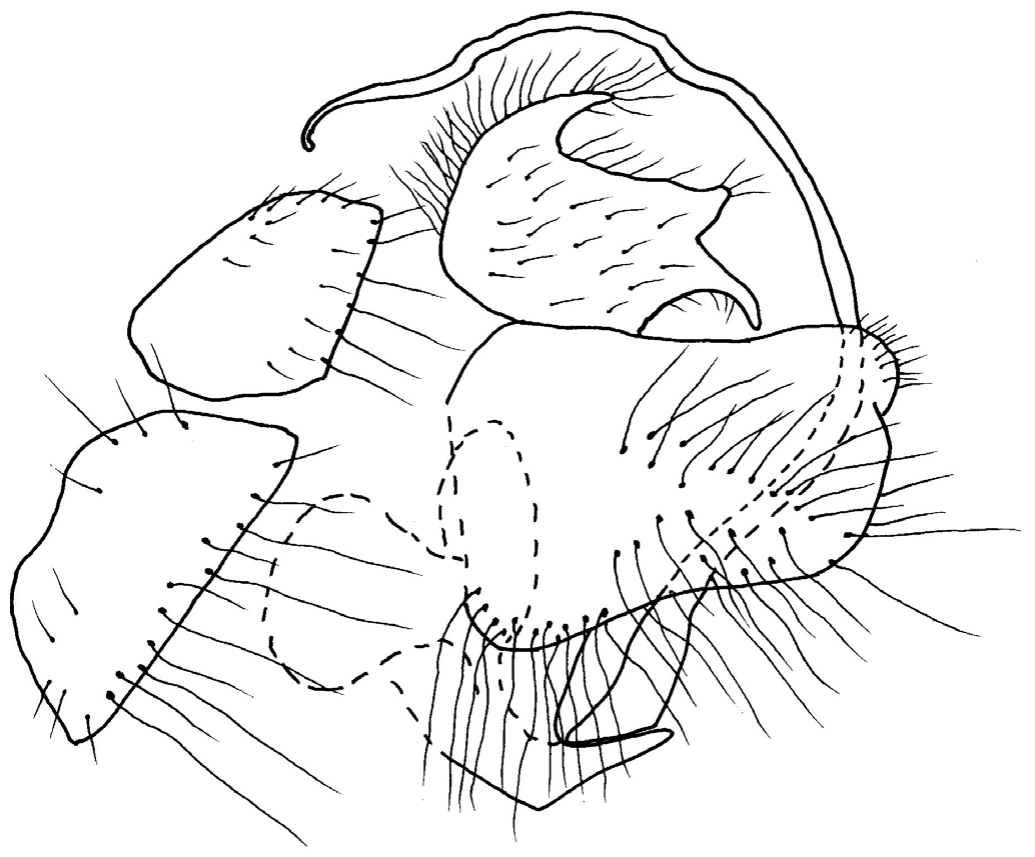
*Empisrhodopensis* sp. n. Male genitalia, lateral view.

**Abdomen** black, light grey microtrichose, lower part of cercus and epandrial lamellae brownish yellow. Tergites dorsally mostly with black setae and lateral parts of tergites and venter mostly with yellow setae. Lateral marginal setae on tergites 2–8 almost as long as segments, discal setae on dorsum of abdomen very short, venter sparsely short setose, with fine hind marginal setae best developed on segments 4–6. Genitalia (Fig. 2): hypandrium small, bare; epandrium subovate, long setose, with slightly swollen dorsoapical part; cercus trilobate with broadly U-shaped dorsal incision; phallus long and thin, slightly undulating apically.

**Length.** body 7.8–8.7 mm, wing 8.5–8.8 mm.

**Female.** Dichoptic, all facets equal in size, frons approx. 0.25 mm broad with approx. 10 short black setae on each side. Labrum 2.8–3.2× longer than head height. Fore femur very short setose. Fore tibia short setose, anterodorsally and posteroventrally with several setae shorter than tibia depth. Mid femur short setose, anteroventrally with extremely short setae (0.03 mm), posteroventrally with row of setae approx. 1/3 of femur depth. Mid tibia with several anterodorsal, posterodorsal and posteroventral setae shorter than tibia depth and with rather dense row of slightly shorter anteroventral setae. Hind femur short setose, ventral setae equally extremely short as those on anteroventral side of mid femur.Hind tibia with several antero- and posterodorsal and anteroventral setae shorter than tibia depth. Tarsi of all legs narrow and short setose, basitarsi with short ventral spines. Abdomen with setae mostly black, yellow ones confined to lateral parts of basal segments.

#### Remarks.

Empis (Leptempis) rhodopensis sp. n. is a member of the E. (L.) rustica group close to E. (L.) trunca Daugeron (subsequently abbreviated ET). However, the newly described species has longer labrum (1.7× head height in ET), brownish wing membrane (clear in ET), mesoscutum with three narrow not very distinct brown stripes along lines of setae (two broad stripes on dorsocentrals in ET), hind femur with strong and short irregularly arranged spines ventrally (absent in ET), epandrial lamellae brownish yellow (brown to black in ET) and phallus longer and thinner (compare figure 8 by Daugeron 1999 with Figure 2). Another species, with which the newly described species should be compared, is E. (L.) lindneri Oldenberg due to similarly very long labrum, however, E. (L.) lindneri has basally undulating phallus and different shape of the cercus. Spinose hind femur is also present in Empis (Leptempis) tenuis Bahid & Daugeron, 2017 however, this species differs from ER in many characters, especially it has quite different genitalia (see Bahid et al. 2017). Interestingly, in CULSP collections there is another still undescribed species of this group close to ET from Turkey, differing chiefly from both all above mentioned species by yellow tarsi.

### Hilara bulgarica

Taxon classificationAnimaliaDipteraEmpididae

Barták sp. n.

http://zoobank.org/EE4F1551-B5FE-457A-9E3E-2E8CD2CF07A1

Figs 3 , 4

#### Type material.

**HOLOTYPE** ♂, **Bulgaria**, Rhodopes Mountains, Pamporovo env., meadow, 41.65N, 24.73E, 1600 m, Barták & Kubík, 22.–24.vi.2016 (CULSP). **PARATYPES**: 16♂, 2♀, same data as holotype; 6♂, 1♀, Rhodopes Mountains, Yundola, 1300 m, pasture 42.063N, 23.855E, Barták & Kubík, 30.vi.2016; 2♂, Rhodopes Mountains, Sniezhanka peak, 1900m, hilltop, 41.637N, 24.680E, Barták & Kubík, 24.–25.vi.2016; 1♂, Rhodopes Mountains, 25km SSW of Plovdiv, 1590 m, meadow, 41.935N, 24.679E, Barták & Kubík, 20.vi.2016; 4♂, 1♀, 9km NEE of Dospat, meadow nr. wood, 1170 m, 41.670N, 24.264E, Barták & Kubík, 23.vi.2016; 2♂, 1♀, Pirin Mountains, 6km SE of Bansko, 1300–1600 m, 41.78N, 23.48E, forest, Barták & Kubík, 1.vii.2016; 3♂, Pirin Mountains, Vikhren hut, 2000 m, alpine meadow, 41.756N, 23.415E, Barták & Kubík, 27.vi.2016 – (CULSP).

#### Diagnosis.

Middle sized species of the *Hilaraintermedia* group with mesoscutum light grey with four dark brown stripes visible in anterodorsal view, changing into one broad dark stripe in posterodorsal view, reaching anterior margin of prescutellar depression (in some specimens median stripe down acrostichals lighter even in posterior view, more conspicuously in females); occiput uniformly dull black and male fore basitarsus extremely enlarged.

#### Etymology.

The species is named after country of its origin (Bulgaria).

#### Description.

**Male head** black, occiput dull black from dorsal view and brownish grey in posterior view with slight indication of somewhat lighter narrowly V-shaped spot behind ocelli; frons wide (0.1 mm broad at narrowest point – approx. twice as broad as anterior ocellus) dull black in dorsal view; rather light grey with dull black margins in anterior view; face light grey and broader than frons, black lateral margins narrower and less conspicuous than those on frons. Eyes with all facets of equal size. Both ocellar and one pair of frontal setae long (0.3 mm – slightly longer than postpedicel), frons with 3–7 pairs of much shorter hairs ventrally and 0–1 pairs of similar hairs dorsally of frontal pair. Postocular row of setae complete, setae black, rather fine and long, with shorter setae behind them, lower part of occiput with rather fine and somewhat longer setae, only several of them just below neck paler. Clypeus and narrow gena entirely almost silvery microtrichose. Palpus black, almost silvery grey microtrichose, usually with 1–2 long and strong ventral setae (longest seta up to 0.47 mm long, approx. as long as labrum) and with many additional fine rather long setae ventrally. Labrum brownish black, polished, 2/3 as long as head height; postmentum nearly as broad and long as labellae with apical circlet of black hairs similar to those on labellae. Antenna black, both basal segments short setose; length of antennal segments (scape: pedicel: postpedicel: 1^st^ segment of stylus: stylus: bare part of stylus) = 0.06–0.08 mm: 0.06–0.08 mm: 0.2–0.25 mm: 0.02–0.03 mm: 0.13–0.18 mm: 0.03–0.04 mm.

**Figure 3.**
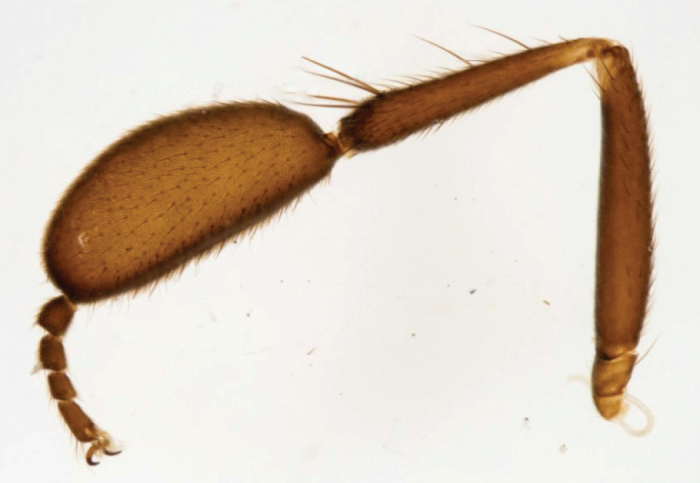
*Hilarabulgarica* sp. n. Male fore leg, posterior view.

**Thorax** black, grey microtrichose, mesoscutum with four dark brown stripes in anterodorsal view (inner pair of stripes between rows of acrostichal and dorsocentral setae narrow, running from antepronotum to posterior acrostichals, outer pair outside dorsocentrals broader), spaces occupied by rows of setae rather brownish grey; in posterodorsal view this pattern changes and mesoscutum except posterior part appears dark almost velvety black (in some specimens median stripe down acrostichals somewhat lighter even in posterior view); prescutellars depression and scutellum very light, and from almost all points of view almost silvery microtrichose; pleuron light (almost bluish) grey. Chaetotaxy: proepisternum with fine pale setulae; prosternum without setae; acrostichals black, irregularly biserial (with tendency to be triserial on middle or posteriorly) and short (approx. 0.09 mm); dorsocentrals uniserial, slightly longer than acrostichals, ending in two pairs of long prescutellars; postpronotal and posthumeral (presutural supraalar) setae long and strong, intrahumeral (presutural intraalar) fine and very short; antepronotum with two strong black setae on sides and 3–5 additional fine but rather long additional setae between them; notopleuron with 2–3 setae (and 6–8 fine yellowish brown setulae on anterior part); one supraalar and one postalar strong setae, prealar region with several setae similar to those on front part of notopleuron; two pairs of scutellars, outer pair shorter. **Legs**: coxae concolorous with pleura, with both yellow and brown setae (smaller setae usually pale, larger darker); more distal parts of legs brownish-black with nearly all setae black, tarsi almost black, microtrichose, knees narrowly yellowish. Fore femur (Figure 3) short setulose, posteroventrally almost bare except several preapicals. Fore tibia apically slightly swollen (at tip subequally deep as femur in middle), dorsally with 2–5 long setae, preapical circlet consists of 4–5 long setae, basitarsus extremely swollen and long, as long as or longer than tibia, rather flattened ventrally, short setulose, tarsomeres 2–4 short, approx. as long as deep. Mid femur short setose, subbasal ventral hair fine and short, anterior setae poorly differentiated. Mid tibia with 1–3 short anteroventral setae and with slightly elongated fine anterodorsal setae slightly longer than tibia depth. Hind femur with dorsal and ventral setae fine and shorter than tibia depth. Hind tibia thin, short setose, 1–3 anteroventral setae shorter than tibia depth, dorsal setae longer than tibia depth, preapical the longest; mid and hind basitarsi ventrally with short spines. **Wings** clear, veins brownish-black, stigma brown, anal vein long, radial fork long, axillary angle obtuse (approx. 130°) costal seta short (approx. as long as outer scutellar). Halter clear yellow, calypter grey with brownish margin and long yellow fringes.

**Figure 4.**
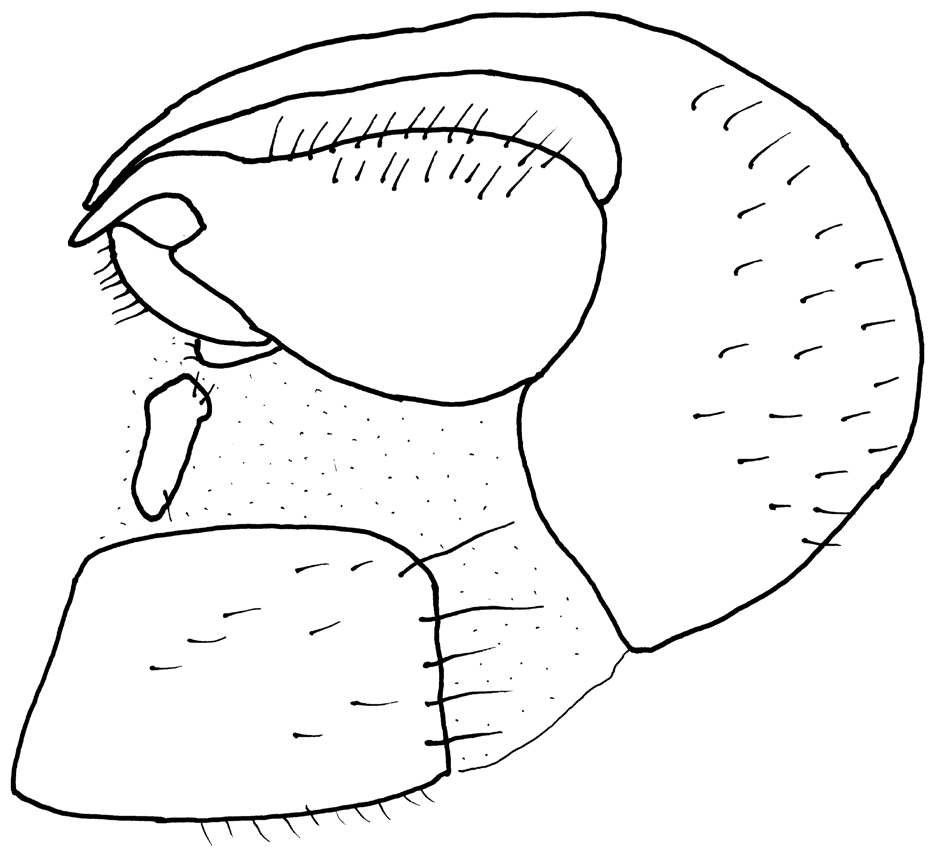
*Hilarabulgarica* sp. n. Male genitalia, lateral view.

**Abdomen** rather long and narrow, brownish-black, very light grey microtrichose, appearing lighter and more brownish than thorax. Lateral marginal setae on tergites 2–6 approx. as long as segments, those on segment 1 and partly (on sides) of segment 2 white to light brownish, on remaining segments black; discal setae very short and black (except somewhat longer whitish setae on segment 2), segment 7 very short setose and marginal setae on segment 8 short (longest setae up to 0.15 mm long, ventral marginal setae not distinctly longer than discal ones); sternites with very short white (on basal segments) to light brownish setae. Genitalia as in Fig. 4: hypandrium almost equally strong nearly to apex; epandrial lamella with narrow and C-shaped apical outgrowth.

**Length.** body 4.1–6.1 mm, wing 4.0–4.7 mm.

**Female.** Very similar to male in all characters except legs. Fore tibia with 2–4 setae dorsally slightly longer than tibia depth. Mid tibia with only 1–2 setae ventrally shorter than tibia depth. Hind tibia practically unmodified, only very indistinctly curved in dorsal view, setae similar as in male but shorter. All tarsi slender and rather long, short setose. Abdomen with shorter marginal setae than in male. **Length.** body 4.0–4.8 mm, wing 3.6–4.1 mm.

#### Remarks.

*Hilarabulgarica* sp. n. is a member of the *H.intermedia* group similar to *H.polleti* Chvála, also described from Bulgaria. Both species clearly differ by the shape of apical process of epandrial lamella, which is rounded and spinose in the latter species. Shape of this process in the newly described species (and four dark thoracic stripes broadly coalescent in posterior view) resembles that of *H.beckeri* Strobl. The latter species differs in many characters, for example, it has quadriserial acrostichals and strongly curved hind tibia in the female. Moreover, the species described above cannot be confused with any other member of the *H.intermedia* group due to extremely enlarged male fore basitarsus.

### Rhamphomyia (Amydroneura) stojanovae

Taxon classificationAnimaliaDipteraEmpididae

Barták sp. n.

http://zoobank.org/E7671FE0-1D14-4AD8-9385-1C874ED47EBA

Figure 5

#### Type material.

**HOLOTYPE** ♂, **Bulgaria**, 5 km W of Smolyan, clearing in wood, 1260 m, 41.569N, 24.632E, Barták, Kubík, 25.vi.2016 (CULSP). **PARATYPES**: 32♂, 9♀, same data as holotype (CULSP).

#### Diagnosis.

Black species of the subgenus Amydroneura with abdomen generally shiny, black setose and with long bow-shaped phallus.

**Etymology**: The species epithet, *stojanovae*, is a Latin genitive patronym to honour Dr. Anelia Stojanova for her help with organizing our expeditions to Bulgaria.

#### Description.

**Male head** black, black setose, narrowly dichoptic. Frons shiny, approx. 0.04 mm wide in middle, slightly widening dorsally, with 4–5 pairs of setae approx. as long as frons is broad. All eye facets approximately of the same size. Ocellar setae black, approx. 0.15 mm long, ocellar triangle microtrichose. Occiput subshiny, only finely microtrichose, with short black setae dorsally and somewhat longer ventrally. Face slightly broader than frons, shiny but somewhat rugose, without setae. Clypeus shiny on anterior half and microtrichose posteriorly, gena very narrow. Palpus black, short, with 5–6 setae approx. 0.10 mm long. Labrum blackish brown, shiny, slightly shorter than head height. Antenna black, both basal segments short setose; length of antennal segments (scape: pedicel: postpedicel: stylus) = 0.04–0.05 mm: 0.06–0.07 mm: 0.24–0.29 mm: 0.06–0.07 mm.

**Figure 5.**
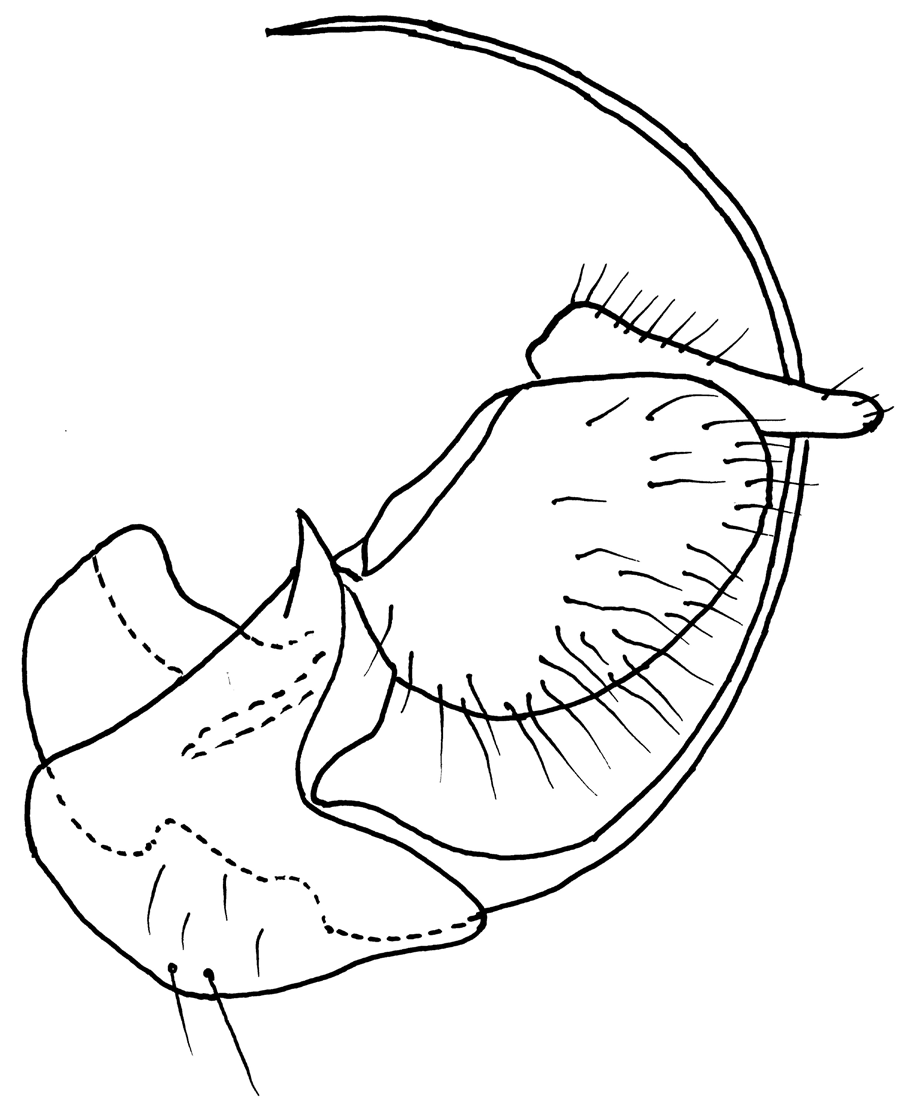
*Rhamphomyiastojanovae* sp. n. Male genitalia, lateral view.

**Thorax** black, mesoscutum subshiny, sparsely microtrichose, more apparent in lateral view, all pleural parts grey microtrichose. All thoracic setae black. Chaetotaxy: antepronotum with several short setae, proepisternum with 1–3 setae, prosternum bare; acrostichals biserial, short (0.05 mm), shorter than distance between rows of acrostichals and dorsocentrals; dorsocentrals equally short, multiserial, spreading out laterally, ending in one long prescutellar pair; posthumeral (presutural supraalar) not differentiated from numerous similar thin setae, intrahumeral (presutural intraalar) present in rather posterior position; postpronotum with 1–2 long, strong setae and several shorter additional setae; notopleuron with 1 long, strong seta accompanied usually with another 1–2 shorter setae and additional smaller and finer setulae; supraalar and prealar regions with several setae similar to those before suture; one long postalar; two pairs of long scutellars; laterotergite black setose. **Legs**: coxae concolorous with pleura; legs brownish black, sparsely microtrichose, black setose. Fore femur with rows of antero- and posteroventral fine setae slightly shorter than femur depth. Fore tibia and tarsus short setose, posterodorsal fine setae slightly elongated, approx. as long as tibia depth. Mid femur with anteroventral row of setae approx. 2/3 as long as femur depth, posteroventrals irregular and short, somewhat longer apically. Mid tibia with short and somewhat stronger setae ventrally, posterodorsally with two setae on basal third almost 3X longer than tibia depth, posterodorsal setosity fine, elongated and almost curled apically, longer than tibia depth, similar setosity also present on mid tarsus. Hind femur rather densely covered with fine setae approx. as long as femur depth, ventral setae slightly shorter and more regularly arranged in row. Hind tibia with ventral ciliation very short, posterodorsally with elongated, fine and almost curled setosity similar to those on mid tibia. Tarsi of all legs with similar setosity as on corresponding tibiae. Seta in comb at tip of hind tibia very short, not longer than setae forming comb. **Wings** clear, slightly iridescent, veins not pigmented, only part of C vein from R_1_ to its end and apical part of vein R_4+5_ somewhat darkened, axillary angle very obtuse; costal seta present, black and long. Measurements: M2/d ratio = 0.7–0.9, CuA1 ratio = 1.7–2.1, lw:ww ratio = 2.4–2.8. Halter brownish black, calypter brown with dark fringes. **Abdomen** black, tergites 2–7 and sternites 1–4 shiny, dorsal parts of tergites finely microtrichose. Lateral marginal setae on tergites 2–6 almost as long as segments, discal setae almost equally long. Genitalia (Figure 5): hypandrium strongly bowed, setose; epandrium subovate, moderately setose; cercus narrow and pointed apically; phallus long and very thin. **Length**: body 2.0–2.3 mm (this species has abdomen at tip bowed ventrally, so actually it is slightly larger), wing 1.9–2.0 mm.

**Female.** Dichoptic, all facets equal in size, frons approx. 0.10 mm broad with 5–6 short black setae on each side. Face broader than frons, shiny anteriorly. Labrum slightly longer than head height. Fore femur with 2 ventral rows of setae at most half as long as femur depth. Fore tibia with dorsal setae scarcely as long as tibia depth. Mid femur short setose, antero- and posteroventrals approx. third as long as femur depth, only somewhat longer preapically.

Mid tibia with submedian posterodorsal seta twice longer than tibia depth and another shorter posterodorsal seta subbasally. Hind femur short setose, ventral setae in middle less than half as long as femur depth. Hind tibia with 2–4 posterodorsal setae slightly longer than tibia depth, otherwise short setose. Tarsi of all legs short setose including basitarsi. Abdomen with tergites 2–6 and sternites 1–5 shiny, tergite 6 subshiny, remaining parts microtrichose. All setae black and very short.

#### Remarks.

Rhamphomyia (Amydroneura) stojanovae sp. n. is most closely allied to *R.claripennis* Oldenberg (RC) and leads to this species in the key by Barták (2006a). However, the male differs in long setulae on basitarsi (short in RC) and different genitalia (with apically broadened cercus and much shorter phallus in RC – see figure 14e by Barták 1982). Distinguishing females is much more difficult. The female leads in the key by Barták (2006a) to *R.claripennis* except tergite 7, which is microtrichose. We found only three characters allowing to distinguish both species: *R.stojanovae* sp. n. has narrower frons (length: width ratio approx. 2) but broader in RC (approx. 1.6), sternite 1 is shiny in RS but microtrichose in RC, and tergite 7 is shiny in RC but microtrichose in RS. *Rhamphomyiabipila* Strobl is another species with which the above described species must be compared. This species, described and still known only from Spain, differs in male, besides many characters (different setosity of legs, longer stylus, narrower frons), by presence of long preapical setae on fore basitarsi and in female by narrower frons (2.5× longer than wide) as well as slightly flattened setae on mid and hind basitarsi.

##### Faunistic records

Empis (Euempis) calcarata Bezzi, 1899. 1♀, Rhodopes Mountains, Pamporovo env., meadow, 1600 m, 41.39N, 24.44E, Barták, Kubík, 22.-24.vi.2016; 1♀, 27 km N of Smolyan, on flowers, nr.river, 770 m, 41.82N, 24.68E, Barták, Kubík, 19.vi.2016; 6♂, 2 km NE of Hristo Danovo, SW, forest path, 1160 m, 42.73N, 24.62E, Barták, Kubík, 15.vi.2017. Remarks: species known from southern parts of the West Palaearctic (Italy, Turkey, Russia – Krasnodarskiy Terr.). These are the first records from Bulgaria.

Empis (Leptempis) discolor Loew, 1856. 7♂, 10♀, Pirin Mountains, 6 km SE of Bansko, 1300–1600 m, 41.78N, 23.48E, forest Barták, Kubík, 1.vii.2016; 1♂, Rhodopes Mountains, Yundola, 1300 m, pasture, 42.06N, 23.85E, Barták, Kubík, 30.vi.2016. Remarks: temperate and south European species. These are the first records from Bulgaria.

Empis (Planempis) frauscheri Linnaeus, 1758. 4♂, 1♀, 9km NEE of Dospat, meadow nr. wood, 1170 m, 41.67N, 24.26E, Barták & Kubík, 23.vi.2016. Remarks: species known only from Romania and Austria. These are the first records from Bulgaria.

Empis (Xanthempis) semicinerea Loew, 1867. 1♂, 1♀, 13 km SW of Troyan, wood+meadow, 1350 m, 42.78N, 24.62E, Barták, Kubík 15.-22.vi.2017; 1♂, 1♀, Pirin Mountains, Bezbog hut, 2200 m, alpine meadow, 41.73N, 23.52E, Barták, Kubík, 28.vi.2016; 1♀, Shipka pass, edge of fagetum, SW, 1240 m, 42.74N, 25.33E, Barták, Kubík 21.vi.2017. Remarks: Central European species.

Empis (Xanthempis) stercorea Linnaeus, 1761. 1♂, Rhodopes Mountains, Pamporovo env., meadow, 1600 m, 41.65N, 24.73E, Barták, Kubík, 22.-24.vi.2016. Remarks: Palaearctic species. This is the first record from Bulgaria.

*Hilaraalbitarsis* von Roser, 1840. 1♂, Rhodopes Mountains, 25 km SSW of Plovdiv, 1590 m, meadow, 41.93N, 24.67E, Barták, Kubík, 20.vi.2016; 2♂, 1♀, Rhodopes Mountains, Pamporovo env., meadow, 1600 m, 41.65N, 24.73E, Barták, Kubík, 22.–24.vi.2016. Remarks: a European species. These are the first records from Bulgaria.

*Hilaraalbiventris* von Roser, 1840. 3♂, Gorno Sahrane, pasture nr.river, sw, 480 m, 42.64N, 25.21E, Barták, Kubík, 18.vi.2017; 1♀, 3 km N of Kalofer, meadow nr.river, sw, 720 m, 42.63N, 24.97E, Barták, Kubík, 16.-26.vi.2017. Remarks: recorded from several European countries ranging from Britain to Romania. These are the first records from Bulgaria.

*Hilaraanglodanica* Lundbeck, 1913. 5♂, 3♀, Koprinka lake, wood, 400 m, 42.62N, 25.28E, Barták, Kubík, 21.vi.2017; 2♂, 3 km N of Kalofer, meadow nr. river, sw, 720 m, 42.63N, 24.97E, Barták, Kubík, 16.-26.vi.2017. Remarks: known from several European countries ranging from Britain to Krasnodarskiy Territory of Russia. These are the first records from Bulgaria.

*Hilaracoracina* Oldenberg, 1916. 1♀, 2 km NE of Hristo Danovo, forest path, 1160 m, 42.73N, 24.62E, Barták, Kubík, 15.vi.2017. Remarks: species distributed in temperate and North Europe. This is the first record from Bulgaria.

*Hilaradiscalis* Chvála, 1997. 1♀, 3 km N of Kalofer, meadow nr. river, sw, 720 m, 42.63N, 24.97E, Barták, Kubík, 16.-26.vi.2017; 1♂, Gorno Sahrane, pasture nr. river, sw, 480 m, 42.64N, 25.21E, Barták, Kubík, 18.vi.2017; 1♂, Pirin Mountains, 6 km SE of Bansko, forest, 1300-1600 m, 41.78N, 23.48E, Barták, Kubík, 1.vii.2016. Remarks: a European species. These are the first records from Bulgaria.

*Hilarafemorella* Zetterstedt, 1842. 3♂, 2♀, Pirin Mountains, Vikhren hut, 2000 m, alpine meadow, 41.75N, 23.41E, Barták, Kubík, 27.vi.2016. Remarks: known from Europe except southernmost parts. These are the first records from Bulgaria.

*Hilarafuscipes* (Fabricius, 1794). 33♂, 13♀, 3 km N of Kalofer, meadow nr. river, sw, 720 m, 42.63N, 24.97E, Barták, Kubík, 16.×26.vi.2017. Remarks: widely distributed Palaearctic species. These are the first records from Bulgaria.

*Hilaragalactoptera* Strobl, 1910. 1♀, Rhodopes Mountains, 25 km SSW of Plovdiv, 1590 m, meadow, 41.93N, 24.67E, Barták, Kubík, 20.vi.2016. Remarks: species known from temperate Europe. This is the first record from Bulgaria.

*Hilaralasiopa* Strobl, 1892. 2♂, Pirin Mountains, 6 km SE of Bansko, forest, 1300-1600 m, 41.78N, 23.48E, Barták, Kubík, 1.vii.2016. Remarks: distributed in several countries in West part of temperate Europe. These are the first records from Bulgaria.

*Hilaralongivittata* Zetterstedt, 1842. 13♂, 2♀, Troyan pass, nr. brook, sw, 1400 m, 42.77N, 24.61E, Barták, Kubík, 22.–24.vi.2017; 3♂, 13 km SW of Troyan, wood+meadow, 1350 m, 42.78N, 24.62E, Barták, Kubík 15.×22.vi.2017; 4♂, Shipka pass, edge of Fagetum, sw, 1240 m, 42.74N, 25.33E, Barták, Kubík, 21.vi.2017. Remarks: Palaearctic species, ranging from Ireland to Far East of Russia, absent from southern parts. These are the first records from Bulgaria.

*Hilaralurida* (Fallén, 1816). 3♂, 3 km N of Kalofer, meadow nr. river, sw, 720 m, 42.63N, 24.97E, Barták, Kubík, 16.-26.vi.2017. Remarks: known from nearly all Europe except southernmost parts. These are the first records from Bulgaria.

*Hilaranigrocincta* de Meijere, 1935. 4♂, Gorno Sahrane, pasture nr. river, sw, 480 m, 42.64N, 25.21E, Barták, Kubík, 18.vi.2017. Remarks: a species of temperate and warm Europe, from the Netherlands, south through central parts of Europe including the Alpine region to the Mediterranean. These are the first records from Bulgaria.

*Hilaranitidorella* Chvála, 1997. 1♂, Troyan pass, nr. brook, sw, 1400 m, 42.77N, 24.61E, Barták, Kubík, 22.–24.vi.2017; 8♂, 1♀, Pirin Mountains, 6 km SE of Bansko, forest, 1300-1600 m, 41.78N, 23.48E, Barták, Kubík, 1.vii.2016. Remarks: distributed from British Isles to Slovenia, northwards to Leningradskaya Province of Russia. These are the first records from Bulgaria.

*Hilaraquadriseta* Collin, 1927. 5♂, 4♀, Rhodopes Mountains, Yundola, 1300 m, pasture, 42.06N, 23.85E, Barták, Kubík, 30.vi.2016; 5♂, Pirin Mountains, 6 km SE of Bansko, forest, 1300–1600 m, 41.78N, 23.48E, Barták, Kubík, 1.vii.2016. Remarks: species known from only several countries of temperate Europe ranging from British Isles to Slovakia. These are the first records from Bulgaria.

*Hilarasplendida* Straka, 1976. 8♂, 3♀, 3 km N of Kalofer, meadow nr. river, sw, 720 m, 42.63N, 24.97E, Barták, Kubík, 16.–26.vi.2017; Rhodopes Mountains, Pamporovo env., meadow, 1600 m, 41.65N, 24.73E, Barták, Kubík, 22.–24.vi.2016. Remarks: known from several European countries ranging from Germany and Switzerland to Romania and the Caucasus (also Turkey – our unpublished data). These are the first records from Bulgaria.

*Hilarasturmii* Wiedemann, 1822. 1♂, 3 km N of Kalofer, meadow nr. river, pt, 730 m, 42.63N, 24.97E, Barták, Kubík, 20.–27.vi.2017. Remarks: widely distributed in Europe from the British Isles and southern parts of Fennoscandia to the Mediterranean, east to Romania. These are the first records from Bulgaria.

*Rhamphomyiaclaripennis* Oldenberg, 1922. 1♂, 27 km N of Smolyan, on flowers, nr.river, 770 m, 41.82N, 24.68E, Barták, Kubík, 19.vi.2016; 1♂, 5 km W of Smolyan, clearing in wood, 1260 m, 41.56N, 24.63E, Barták, Kubík, 25.vi.2016; 1♂, Rhodopes Mountains, 25 km SSW of Plovdiv, 1590 m, meadow, 41.93N, 24.67E, Barták, Kubík, 20.vi.2016. Remarks: previously known from only several countries ranging from Germany to Italy. These are the first records from Bulgaria.

*Rhamphomyiacrinita* Becker, 1887. 10♂, 3♀, Pirin Mountains, Vikhren hut, 2000 m, alpine meadow, 41.75N, 23.41E, Barták, Kubík, 27.vi.2016. Remarks: species distributed in mountains of Central Europe. Possibly complex of siblings. These are the first records from Bulgaria.

*Rhamphomyiadudai* Oldenberg, 1927. 1♂, 5 km W of Smolyan, clearing in wood, 1260 m, 41.56N, 24.63E, Barták, Kubík, 25.vi.2016. Remarks: species known from most parts of temperate and North Europe. This is the first record from Bulgaria and, contemporarily, southernmost distributional record.

*Rhamphomyiamagellensis* Frey, 1922. 3♂, 2♀, Pirin Mountains, 6 km SE of Bansko, forest, 1300–1600 m, 41.78N, 23.48E, Barták, Kubík, 1.vii.2016. Remarks: species known from several European mountains ranging from Pyrenees to Slovakia. These are the first records from Bulgaria and, contemporarily, easternmost distributional records.

*Rhamphomyianudipes* Oldenberg, 1927. 1♂, Rhodopes Mountains, 25 km SSW of Plovdiv, 1590 m, meadow, 41.93N, 24.67E, Barták, Kubík, 20.vi.2016; 1♂, Shipka pass, edge of Fagetum, sw, 1240 m, 42.74N, 25.33E, Barták, Kubík, 21.vi.2017. Remarks: reported only from Italy but we have other (unpublished) records from France, Austria and Greece. These are the first records from Bulgaria.

*Rhamphomyiasphenoptera* Loew, 1873. 2♂, Pirin Mountains, Vikhren hut, 2000 m, alpine meadow, 41.75N, 23.41E, Barták, Kubík, 27.vi.2016. Remarks: known with certainty from Italy, Serbia and Montenegro, and Albania. Records from Greece and Hungary need verification. These are the first records from Bulgaria.

*Rhamphomyiaumbripennis* Meigen, 1822. 1♂, Pirin Mountains, Bezbog hut, 2200 m, alpine meadow, 41.73N, 23.52E, Barták, Kubík, 28.vi.2016. Remarks: species known from most of Europe except southernmost parts. This is the first record from Bulgaria.

*Rhamphomyiaumbripes* Becker, 1887. 1♂, 13 km SW of Troyan, wood+meadow, 1350 m, 42.78N, 24.62E, Barták, Kubík 15.–22.vi.2017. Remarks: known only from several central European countries (Germany, Czech Republic, Slovakia, Austria, and Italy). Recently Chvála and Pont (2015) synonymised the subgenus Aclonempis Collin, 1926 (genus *Rhamphomyia*) with the genus *Empis* but at this moment (prior to complete re-classification of the whole tribe Empidini) this is not accepted here. This is the first record from Bulgaria.

## Discussion

The degree of knowledge of Empididae from Bulgaria has been very low. Altogether 106 species have been previously published from this country but several published records remain doubtful (including *Rhamphomyia simplex, R. lamelliseta*). An additional study is necessary to elucidate their identity.

In this paper, Empis (Leptempis) rhodopensis sp. n., *Hilarabulgarica* sp. n., and Rhamphomyia (Amydroneura) stojanovae sp. n. are described from Bulgaria as new species and 29 species are first reported from this country. These findings increased the total number of Bulgarian Empididae to 138.

## Supplementary Material

XML Treatment for Empis (Leptempis) rhodopensis

XML Treatment for Hilara bulgarica

XML Treatment for Rhamphomyia (Amydroneura) stojanovae
